# Supplementary material for: Double Perovskite LaFe1−xNixO3 Coated with Sea Urchin-like Gold Nanoparticles Using Electrophoresis as the Photoelectrochemical Electrode to Enhance H2 Production via Surface Plasmon Resonance Effect
Source: Nanomaterials (Basel). 2022 Feb 12;12(4):622. doi: 10.3390/nano12040622 (PMC8876238; doi:10.3390/nano12040622)
Supplement: Supplementary file 1 [file nanomaterials-12-00622-s001.zip › nanomaterials-1592449-supplementary.pdf]

nanomaterials

MDPI

Supplementary Material

Double Perovskite $LaFe_{1-x}Ni_xO_3$ Coated with Sea Urchin-like Gold Nanoparticles Using Electrophoresis as the Photoelectrochemical Electrode to Enhance H_2 Production via Surface Plasmon Resonance Effect

Hsiang-Wei Tsai and Yen-Hsun Su *

Department of Materials Science and Engineering, National Cheng-Kung University, 701401, Taiwan; n58064026@gs.ncku.edu.tw
* Correspondence: yhsu@ncku.edu.tw; Tel.: +886-6-275-7575 (ext. 62941)

Table S1. Particle sizes of the raw LFNO samples with different Ni concentrations.

Sample	Size (μm)
0.00 mole	4.72 ± 0.34
0.01 mole	7.25 ± 0.80
0.03 mole	7.35 ± 0.85
0.05 mole	7.63 ± 0.81
0.07 mole	7.82 ± 0.80

Table S2. UV-vis properties of the raw and Au-coated LFNO samples with different Ni concentrations.

Sample	LFNO		Au-coated LFNO	
	E_g (eV)	Wavelength (μm)	PE (eV)	Wavelength (μm)
0.00 mol	2.21	561.1	2.08	596.2
0.01 mol	2.20	563.6	2.05	604.9
0.03 mol	2.12	584.9	2.04	607.8
0.05 mol	2.09	593.3	2.02	613.9
0.07 mol	2.05	604.9	1.99	623.1

Table S3. Electrochemical properties of the raw LFNO samples with different Ni concentrations in 0.1 M KOH solution.

Sample	E_p^a (V)	E_p^c (V)	HER (V)
0.00 mol	0.003	−0.002	0.83
0.01 mol	0.368	−0.286	0.67
0.03 mol	0.194	−0.239	0.66
0.05 mol	0.272	−0.318	0.71
0.07 mol	0.156	−0.475	0.82

Table S4. Hydrogen efficiency and real production of the Au-coated LFNO samples with different Ni concentrations in ethanol under AM 1.5 G at different temperatures.

Sample	0 °C			
	V_{op} (V)	I_{op} (×10^{-4} mA)	η (×10^{-3} %)	Real production (μmol $g^{-1}h^{-1}$)
0.00 mol	0.002	1.12	1.37	4478.0

Sample	V_{op} (V)	I_{op} ($\times10^{-4}$ mA)	η ($\times10^{-2}$ %)	Real production (μmol $g^{-1}h^{-1}$)
0.01 mol	0.004	1.08	1.32	4314.6
0.03 mol	0.009	1.05	1.29	4216.5
0.05 mol	0.005	1.05	1.28	4183.8
0.07 mol	0.002	1.09	1.34	4380.0
		**10 °C**		
**Sample**	**V_{op} (V)**	**I_{op} ($\times10^{-4}$ mA)**	**η ($\times10^{-2}$ %)**	**Real production (μmol $g^{-1}h^{-1}$)**
0.00 mol	0.012	1.05	1.28	41838.4
0.01 mol	0.014	1.10	1.34	43800.0
0.03 mol	0.003	0.99	1.22	39877.2
0.05 mol	0.005	1.06	1.30	42492.1
0.07 mol	0.004	1.04	1.28	41838.4
		**20 °C**		
**Sample**	**V_{op} (V)**	**I_{op} ($\times10^{-4}$ mA)**	**η ($\times10^{-2}$ %)**	**Real production (μmol $g^{-1}h^{-1}$)**
0.00 mol	0.005	1.02	1.25	40857.8
0.01 mol	0.004	1.01	1.24	40530.9
0.03 mol	0.002	1.00	1.22	39877.2
0.05 mol	0.002	0.97	1.19	38896.6
0.07 mol	0.004	1.02	1.25	40857.8
		**30 °C**		
**Sample**	**V_{op} (V)**	**I_{op} ($\times10^{-4}$ mA)**	**η ($\times10^{-2}$ %)**	**Real production (μmol $g^{-1}h^{-1}$)**
0.00 mol	0.002	1.00	1.22	39877.2
0.01 mol	0.001	0.97	1.20	39223.5
0.03 mol	0.008	0.97	1.20	39223.5
0.05 mol	0.002	0.96	1.18	38569.7
0.07 mol	0.012	0.98	1.20	39223.5

Table S5. Hydrogen efficiency and real production of the Au-coated LFNO samples with different Ni concentrations in 1-butanol under AM 1.5G at different temperatures.

		**0 °C**		
**Sample**	**V_{op} (V)**	**I_{op} ($\times10^{-4}$ mA)**	**η ($\times10^{-3}$ %)**	**Real production (μmol $g^{-1}h^{-1}$)**
0.00 mol	0.001	9.21	1.13	3693.5
0.01 mol	0.002	9.17	1.13	3693.5
0.03 mol	0.001	9.36	1.15	3759.9
0.05 mol	0.002	9.15	1.12	3660.9
0.07 mol	0.010	9.53	1.16	3791.6
		**10 °C**		
**Sample**	**V_{op} (V)**	**I_{op} ($\times10^{-4}$ mA)**	**η ($\times10^{-3}$ %)**	**Real production (μmol $g^{-1}h^{-1}$)**
0.00 mol	0.004	9.15	1.12	3660.9
0.01 mol	0.007	9.51	1.16	3791.6
0.03 mol	0.003	9.14	1.12	3660.9
0.05 mol	0.004	9.33	1.14	3726.2
0.07 mol	0.011	9.14	1.11	3628.2
		**20 °C**		
**Sample**	**V_{op} (V)**	**I_{op}**	**η**	**Real production**

		(×10^{-4} mA)	(×10^{-3} %)	(μmol $g^{-1}h^{-1}$)
0.00 mol	0.005	9.24	1.13	3693.5
0.01 mol	0.020	9.20	1.11	3628.2
0.03 mol	0.010	9.27	1.13	3693.5
0.05 mol	0.005	9.16	1.13	3693.5
0.07 mol	0.002	9.06	1.11	3628.2
			**30 °C**	
**Sample**	**V_{op} (V)**	**I_{op} (×10^{-4} mA)**	**η (×10^{-3} %)**	**Real production (μmol $g^{-1}h^{-1}$)**
0.00 mol	0.008	8.95	1.09	3562.8
0.01 mol	0.005	9.17	1.12	3660.9
0.03 mol	0.006	8.87	1.10	3595.5
0.05 mol	0.003	8.95	1.10	3595.5
0.07 mol	0.008	8.95	1.09	3562.8

Table S6. Activation energy, E_a, of the Au-coated LFNO samples with different Ni concentrations in ethanol and 1-butanol.

**Sample**	**E_a (eV)**	
	**ethanol**	**1-butanol**
0.00 mol	−0.080	−0.056
0.01 mol	−0.068	−0.055
0.03 mol	−0.063	−0.059
0.05 mol	−0.068	−0.057
0.07 mol	−0.065	−0.058

Table S7. Parameters of the energy-level diagram (vs. vacuum) of the raw LFNO samples with different Ni concentrations.

**Sample**	**E_g (eV)**	**E_{VB} (eV)**	**E_{CB} (eV)**	**E_{FB} (eV)**	**Carrier density (× 10^{19} / cm^3)**
0.00 mol	2.21	−6.18	−3.97	−4.50	4.33
0.01 mol	2.20	−6.32	−4.12	−4.31	3.70
0.03 mol	2.12	−5.96	−3.84	−4.39	3.30
0.05 mol	2.09	−5.97	−3.88	−4.58	4.03
0.07 mol	2.05	−6.08	−4.03	−4.69	4.76
